# Neonatal Nurses’ Understanding of the Factors That Enhance and Hinder Early Communication Between Preterm Infants and Their Parents: A Narrative Inquiry Study

**DOI:** 10.1111/1460-6984.70093

**Published:** 2025-07-14

**Authors:** Julia Petty, Lisa Whiting, Celia Harding

**Affiliations:** ^1^ School of Health, Medicine and Life Sciences University of Hertfordshire Hatfield, England UK; ^2^ Division of Language and Communication Science St. Georges & City University of London London, England UK

**Keywords:** early communication, narrative inquiry, nursing, parents, preterm infants

## Abstract

**Background:**

Infants born preterm are at high risk of facing difficulties with acquiring speech, language and communication skills. Research on the direct benefits of parent–infant communication in neonatal units is limited. This study recognises that although neonatal nurses regard early communication as important, there is scope to develop a wider range of resources to help support professionals’ understanding of the importance of communication in neonatal care.

**Aim:**

To explore neonatal nurses’ understanding of factors that can enable or hinder early communication and interaction between preterm infants and parents within a neonatal unit setting.

**Methods and Procedures:**

This study employed a narrative inquiry approach with nine neonatal nurses, selected through purposive sampling. Narrative interviews investigated nurses’ views and understanding of the enablers and challenges to communication in this patient group, along with their role in enhancing early communication between infants and parents. Data reporting was undertaken using the Consolidated Criteria for Reporting Qualitative Studies (COREQ), aligning with Enhancing the QUAlity and Transparency of Health Research (EQUATOR) framework.

**Outcomes and Results:**

Narrative analysis revealed the following four themes: the importance of education and experience in neonatal care; supporting parents of infants receiving neonatal care; encouraging communication strategies; the impact of limiting parental presence and wearing facemasks.

**Conclusions and Implications:**

Neonatal nurses commented that using early communication strategies with infants and supporting parents to learn how to communicate directly with their infant is essential. However, none were able to fully describe the key components of early communication from a linguistic perspective, nor give specific examples beyond skin‐to‐skin care, bonding, reading infant cues and hearing familial voices during conversation and reading. Although these are very important antecedent skills that provide a framework for developing communication, they are not always a direct means to enhance language development specifically. As preterm infants are at high risk of altered language and communication development, a real need exists for neonatal nurses to develop linguistically rooted methods to support communication for parents and their infants, in conjunction with allied healthcare professionals such as speech and language therapists. This support can enable the development of positive communication, enriched and extended after leaving the neonatal unit.

**WHAT THIS PAPER ADDS:**

*What is already known on the subject*

- Encouraging and supporting parents to learn to develop early communication and interaction skills with their preterm infants, when experiencing neonatal care, is recognised as being important; previous research has clearly identified these infants as being at high risk of speech, language and communication difficulties. These complications, if they arise, are known to impact future outcomes, including educational achievements along with social and friendship skills. Neonatal nurses in conjunction with key allied healthcare professionals such as speech and language therapists are in a prime position to support parents in this vital area of care.

*What this paper adds to existing knowledge*

- Our study highlights that although neonatal nurses regard early communication as an important part of their role, it identifies a need for them to be further informed and educated about the specifics of early communication, the associated risks and the potential impact on language that poor, unenriched communication environments can lead to. Importantly, although some studies have investigated aspects of early preterm infant–parent interaction, there is still a need for further in‐depth investigation into early parent communication confidence and skills, particularly in relation to supporting linguistic development by neonatal nurses and allied healthcare professionals, including speech and language therapists.

*What are the potential or actual clinical implications of this work?*

- Collaboration between neonatal nurses and other professionals, in particular speech and language therapists, is essential so that preterm infants and their families can experience language‐rich learning environments within a neonatal unit; parents can then be supported to use tailored strategies to enable positive language experiences when discharged home. Speech and language therapists working with neonatal nurses can substantially enhance the quality of infant–parent interactions in neonatal environments through direct modelling, education and the creation of resources for both families and neonatal staff, which includes accurate information related to a linguistic framework, on which future work will focus and investigate further.

## 1 Introduction

Infants born below 37 weeks gestation are identified as preterm, often requiring complex neonatal nursing care and support (Akkoyun and Tas Arslan 2019). Coping with an unfamiliar neonatal environment can have a negative impact on parental mental health and delay the confident acquisition of parental skills to care for their own infant (Maleki et al. 2022). Nurses who work in neonatal care encourage parents to develop their caring abilities and to experience both physical and emotional closeness with infants so that bonding can develop (Gibbs et al. 2021). Approaches that nurses recommend, such as interpreting infant cues and developing attachment through care and skin‐to‐skin time, are often described as methods of ‘communication’. These strategies, if shaped, can be preparatory procedures that support the development of positive dyadic relationships, which can be built on to promote interactive and mutually enjoyable linguistic exchanges (Harding et al. 2019, 2022). Communication is learning to understand language, facial expression, gesture and tone of voice as well as developing the skills to become a communication partner during interactions by using gestures, facial expressions, and vocalisations (Harding et al. 2019). Speech, language and communication difficulties are a known developmental risk for infants born preterm; encouraging carers to engage in early, meaningful linguistic interaction can enable the development of long‐term language‐rich environments (Aylward 2014; Harding et al. 2019; Iverson and Goldin‐Meadow 2005; Rabie et al. 2015; Sanchez et al. 2019; Sansavini et al. 2011; Serenius et al. 2013; van Noort‐van der Spek et al. 2012; Wolke et al. 2008). More specifically, infants born preterm are at higher risk of being slow to acquire natural gestures that accompany language development, acquiring first words and understanding vocabulary (Sansavini et al. 2011). Gestures are an important developmental support that can enable vocabulary consolidation when learning first words, but at 12–18 months, children born preterm use fewer gestures with their communication attempts, thereby curtailing language acquisition progression (Cattani et al. 2010). Although infants born preterm can develop functional gestures and increase usage over time, there are fewer deictic gestures used, and this impacts sustained interactions with others (Suttora and Salerni 2012). For very low birthweight infants, greater risks are associated with smaller receptive language vocabularies, difficulties learning words and a lower number of learnt words that are functionally used in day‐to‐day interactions at 12 months of age (Stolt et al. 2009). Compared with typically developing peers, preterm infants are at risk of developing shorter utterances, smaller expressive vocabularies and challenges of combining words (Félix et al. 2017; Sentenac et al. 2020).

Healthcare professionals, in particular speech and language therapists working with neonatal nurses, have an important role in providing communication support within neonatal units so that there is sustained synchronous development between parents and infants (Gibbs et al. 2021). Within the literature, communication is still misunderstood and misrepresented with discussion of language and interaction with infants being used interchangeably with parent–neonatal staff interaction and dynamics (Guillaume et al. 2013).

## 2 Background

Neonatal nurses provide direct healthcare interventions for infants and support parents to gain skills so they can bond with their child and develop confidence when providing care (Gibbs et al. 2021). In a variable, unfamiliar and challenging environment, promoting early infant learning can be compromised by inconsistent advice and parent support from nursing staff (Maleki et al. 2022). In collaboration with other neonatal professionals, guidance for parents is an essential part of the nursing role as the quality of early infant–parent interactions influences subsequent care and infant management (Muller‐Nix et al. 2004). Factors such as unaddressed parent depression and trauma can reduce positive infant–parent experiences; nurses, therefore, have a key role in identifying parents who are struggling (Korja et al. 2008). The COVID‐19 pandemic resulted in reduced access to the cot‐side, creating increased parental anxiety and stress that was further complicated by usage of facemasks and personal protective equipment (PPE), thereby restricting early communication skill acquisition, such as facial recognition and gaze (Green et al. 2021; Harding et al. 2022).

The day‐to‐day noise within neonatal units can present challenges for staff and can impact effective working with traumatised families (Cardoso et al. 2015). Noise exposure from monitors, respiratory equipment, alarms as well as staff talking to others at the cot‐side can inhibit or reduce positive and reciprocal language exposure for infants (Cardoso et al. 2015; Lindberg and Öhrling 2008). Parental presence is important for preterm infant communication development as parents use a greater amount of vocal interaction with their infant compared to nursing staff (Caskey et al. 2014; Zwimpfer et al. 2021).

Some parents have been encouraged to use early communication strategies with their infant in a neonatal unit, although support is variable with inconsistent advice about the actual components of communication and interaction (Petty et al. 2024). Parents value guidance in terms of how to communicate with their infant, and such help can provide a skill set that can be built on further when developing speech, language and communication post‐discharge from the neonatal unit (Guillaume et al. 2013; Harding et al. 2022). Stress, depression and trauma related to experiencing a preterm birth prevent positive communication between a parent and baby from developing, but with support, mothers have been observed to be more responsive when interacting once feelings of stress have reduced (Milgrom et al. 2013). Use of voices, including variation of tone by family members, can help with physiological stability (Saliba et al. 2018). Carer use of targeted spoken words and simple phrases directed towards their infants, by talking directly to them whilst receiving neonatal care, enables infants to vocalize more; this leads to subsequent improved language and cognitive outcomes at 7 and 18 months compared with control group peers (Caskey et al. 2014). Families who receive advice about promoting a robust and meaningful language environment can maintain the strategies learnt in the neonatal unit post‐discharge (Milgrom et al. 2013). Infants whose health is improving, and who are no longer requiring an incubator, develop an increased interest in others; parents react accordingly by use of longer periods of sustained eye‐contact and talking (Coppola and Cassibba 2010).

### 2.1 Purpose

Support for parents to develop dyadic synchrony is essential for language development (Coppola and Cassibba 2010). Encouraging parents and key staff to use specific early language and communication skills on a neonatal unit is important and necessary (Harding et al. 2019). However, little is known about neonatal nursing knowledge in this area; therefore, the aim of this study was to explore neonatal nurses’ understanding of factors that can enable or hinder early communication and interaction between preterm infants and parents within a neonatal unit setting.

## 3 MATERIALS AND METHODS

### 3.1 Procedure

This study drew on a narrative inquiry approach (Holloway and Galvin 2016) using narrative interviews to investigate our stated aim. Narrative inquiry, developed initially within education by Clandinin and Connelly (2000), focuses on exploring experiences as described by those who have lived them. It was designed to explore and understand real‐life experiences through the eyes of research participants, allowing a rich description of these experiences and an exploration of the meanings behind them. It uses personal events as a way of communicating the participants' stories to a larger audience (Reissman 2008) providing an in‐depth understanding of their viewpoints as applied to their own context (Trahar 2013). In this case, the context is the experience of working in neonatal nursing care over a trajectory of time.

This methodology was ideally suited for our study as we were seeking the perspectives of neonatal nurses and how this information could enhance practice. Narrative inquiry especially focuses on co‐construction of the participants ‘story’ over time and as a methodology in nursing research is very useful to uncover nuance and detail (Wang and Geale 2015). Narrative inquirers are less concerned with factual participant accounts, but rather in the meanings portrayed in story form (Floersch et al 2010; Holloway and Galvin 2016); interviews may use prompts to encourage participants to recount their experience over a particular time period, but compulsory or structured questions are not used. Participants construct their stories to convey a specific perspective (Haydon et al. 2018) brought out by unstructured interviewing, as was the case in our study, that is, neonatal nurses' experiences of communication between themselves and families in their care as well as their role in supporting parents to interact with their preterm infants. Parent views were also sought using the same design and methodology, as a distinct data set, and are the focus of another separate paper (Petty et al. 2024).

In summary, the need to understand the neonatal nursing experience over time and within an individual context led to the team agreeing that a qualitative, narrative inquiry interview approach was preferable; the use of words such as ‘how’, enabled us to address our research aim and gain the valuable insight that we were seeking, for example, *Thinking about your time working on the neonatal unit, could you tell me* ***how*** *you have supported parents to communicate and interact with their preterm babies?* (see appendix).

### 3.2 Recruitment

Prior to undertaking the study, a Patient Public Involvement (PPI) group was established comprising three parents of preterm infants. The research team shared project aims, recruitment methods, proposed interview topic guide, participant information sheets and asked for comments; minor amendments were made. Appropriate ethics approval was gained from the UK Health Research Authority as well as adherence to Trust R&D processes and policies where the research was undertaken (IRAS ID: 309641).

Nurse participants were recruited from two neonatal units (a Levels 1 and 2) in an outer‐city United Kingdom (UK) district. All nursing staff, across both sites, who had direct contact with infants delivering care on the neonatal units were eligible to be recruited. Participants had to be employees of the National Health Service [NHS] Foundation Trust where the research took place and able to give valid consent in accordance with the Mental Capacity Act (2005). Exclusion criteria were nurses who were not permanent employees of the NHS Foundation Trust, were only involved in infant transfers, did not undertake direct care of infants on the unit, and those who had recent experience of having a preterm infant themselves, as their parental perspective may have influenced their responses to interview questions.

Potential nurse participants were informed of the study through flyers, emails and staff meetings with participant information sheets being provided; these included explanations of the aims, methods, anticipated benefits and potential hazards of the study; nurses could request further discussions with the research team before electing to take part. A dedicated research email account was set up for communication between the research team, the neonatal unit gatekeepers and the volunteer participants. This was clearly visible on the recruitment flyer and all study documentation. All potential participants were reassured that they were under no obligation to take part; those who elected to do so were provided with an external contact, should they wish to raise concerns about the conduct of the study, were informed that they could withdraw at any time without being penalised; all gave signed consent before their interview.

### 3.3 Measures and Procedures

A self‐selecting sample of nine neonatal nursing participants was recruited and interviewed (Table 1). Participants all identified as female, had between 2 and 32 years of nursing experience and had spent 2–25 years working on a neonatal unit. The age range of the participants was 23 to >50 years and encompassed a range of ethnicities (*n* = 4 White British, *n* = 1 Black British, *n* = 1 White Spanish, *n* = 2 Indian, *n* = 1 White Albanian). Previous neonatal communication‐specific training/education amounted to no more than 2–3 days. No participant requested that their data be withdrawn from the study.

**TABLE 1:** Participant details.

Nurse [N]	Age (yrs.)	Ethnicity (as described by participant)	Gender M/F/NB/O PNTS	Quals	Years qualified	Years in neonatal care	Grade and job title	Previous training in communication
N1	33	Black British	F	Adult nurse BSc	12	9	Band 5 Staff nurse	No
N2	26	White Spanish	F	Adult nurse BSc plus QIS	3	3	Band 5 Staff nurse	Yes—Half a day
N3	23	White British	F	Child nurse plus QIS	2	2	Band 5 Staff nurse	Yes 2–3 days
N4	30	White British	F	Child nurse plus QIS	4	3	Band 6 Junior sister	Yes 3 days
N5	38	Albanian	F	Child nurse plus QIS	10	10	Band 6 Discharge nurse	Yes Part of a module—1 day
N6	36	Indian	F	Adult nurse BSc plus QIS	10	8	Band 6 Junior sister	No
N7	32	White British	F	Child nurse BSc plus QIS	9	9	Band 6 Junior sister + FiCare nurse	Yes Half day as part of FiCare course
N8	>50	White British	F	Adult nurse BSc plus QIS	32	25	Band 6 Community sister	Yes As part of two courses—1.5 days
N9	31	Indian	F	Adult nurse BSc	2.5	2.5	Band 5 Staff nurse	No

Abbreviations: F, female; FiCare, Family Integrated Care; M, male; NB, non‐binary; O, other; PNTS, prefer not to say; QIS, qualified in (neonatal) specialty.

All nine narrative interviews were undertaken by one nurse researcher in the team, who had no previous employment within either of the units or the NHS Trust and was not known to the participants. The interviews were between 25 and 60 min in length and were conducted via videoconferencing (Zoom) and digital audio recording. This approach was used to facilitate a broader reach across shift and rota patterns and was welcomed by the participants. A transcript of the interview was produced by a professional company. Where appropriate, the topic guide (see appendix) was referred to as a prompt. Each participant took part in one interview only. Transcripts were assigned a code, and all data were de‐identified. Written records of participants’ names and consent forms were kept separately from the research data. All data were stored on a secure password‐protected University OneDrive folder.

### 3.4 Analysis

Narrative analysis (Holloway and Galvin 2016), as described by Reissman (2008) and Floersch et al. (2010), was used. This approach differs from just using a thematic approach, which identifies recurring patterns or themes. Narrative analysis interprets participants’ stories holistically, emphasising temporality, context and relationships (Haydon et al. 2018). However, the team viewed both methods as essential: we aimed to explore nurses’ stories openly, while also identifying themes useful for future resource development. Therefore, in common with others (Floersch et al. 2010; Petty et al. 2019; Zelčāne and Pipere 2023), we used a combination of narrative and thematic analysis. This hybrid stance aligned with Crossley's (2000) six‐step framework: (1) reading and familiarizing, (2) identifying key concepts, (3) identifying narrative tone through meaningful statements, (4) identifying themes, (5) weaving the analysis together and (6) writing the report.

Each transcript was read several times by the research team. Meaningful statements were identified and coded. Two team members who had not conducted the interviews explored similarities and differences in the nurses’ accounts. Analysis software was considered as this can improve auditability and support collaborative coding, potentially enhancing rigour and trustworthiness. However, this was weighed against concerns of potential disengagement from the data, where focus may shift from meaning to technique (Clarke et al. 2021). With our small dataset, manual coding was deemed more practical and better suited to our inductive approach; hence, software was not used. Inductive coding, rather than deductive, allowed themes and patterns to emerge naturally, without pre‐defined categories. Codes were organized into 10 preliminary categories (Floersch et al. 2010), namely, educational trajectory, previous neonatal experience to date, neonatal nursing role, supporting parents, communication strategies, enablers, barriers, including the impact of restricting parental presence and effects of mask‐wearing and learning resources.

The three researchers reviewed and refined these categories after re‐reading the transcripts two to three times to improve familiarity and rigour, ultimately reaching consensus on the final themes. Additional strategies to enhance rigour included a clear study aim, documented research meetings, a reflexive approach throughout, and maintaining a detailed audit trail. Data reporting followed the Consolidated Criteria for Reporting Qualitative Studies (COREQ), aligning with the EQUATOR framework. Participants were offered the option to validate the themes. Only two chose to do so, but the final report was shared with all participants.

## 4 Results

From the preliminary categories identified, four main themes were agreed upon by the team, namely, the importance of nurse education and experience in neonatal care; supporting parents whose infants were receiving neonatal care; encouraging communication strategies; the impact of limiting parental presence and wearing facemasks. These themes came from the descriptions from the participants relating to temporality (their neonatal career trajectory) and place/contact (neonatal care speciality); by this, we acknowledged the lived experience of participants as a co‐constructed knowledge source (Pino Gavidia and Adu 2022), a key tenet of narrative inquiry.

1. The importance of nurse education and experience in neonatal care: ‘*Always I find it joyful*’ [**N1**]

In line with a narrative approach, all participants spoke about their neonatal career trajectory and learning journey to date, stated that they had received a wide variety of post‐qualification neonatal education, some of which was externally provided and some internally by the relevant NHS Trust (Table 1). When specifically asked about communication‐related education, some nurses mentioned newborn behavioural observation programmes. None had taken part in any courses that focused on early preverbal communication and the key strategies that are essential to the development of functional speech and language. None of the participants knew of any specific training available in this area.

Participants discussed key aspects of their work and nursing role throughout their career to date; facilitating communication between families and their infants was regarded as a fundamental part of their work **[N1, N4]**. Participants spoke of their experiences in this area as valuable and rewarding to themselves personally; they felt privileged and often emotionally moved by being able to facilitate connections between parents and their vulnerable infant. Another separate but intricately linked facet of neonatal unit communication (highlighted by all participants) was that between staff and parents, seen as a vital part of their neonatal nursing role. This applied to their daily work with families and was seen as important and essential, while recognising the barriers that could influence this (for example, busy unit activity, multiple tasks, lack of time). One nurse had a specific role where she spent time working with and supporting families from a ‘Family Integrated Care’ [known as ‘FiCare’] perspective alongside her neonatal nursing role: *‘So I have a couple of management days a month at the moment where that's just solely based on that FiCare….engage with the parents… I think we think that we know what we're doing sometimes in terms of making things better, but actually hearing from the parents is where you're going’* **[N7]**.

The importance of communicating key information to parents was also recognised as a crucial area of their nursing role, throughout their whole neonatal journey and in conjunction with the care team: ***‘*** *They often ask us about equipment and what they need for babies to progress, and it's trying to and initially for us is explaining that actually, what they need is you’* **[N8]**. Regarding the multi‐disciplinary team, participants valued the knowledge acquisition from a variety of allied healthcare professionals, particularly speech and language therapists and occupational therapists, who contributed significantly to their ability to support parents with communication and emotional support.

2. Supporting parents whose infants were receiving neonatal care: ‘Very difficult, then, isn't it, to find that time to give that emotional support, so it's not something you can just go in and do quickly, and then leave’ [**N7**]

This theme generated examples of support that nurses spoke openly about in the course of their work, which they felt encouraged parents to develop close and nurturing connections with their infants. A strong presence within the participant narratives was the recognition of the emotional stress of having a preterm infant for families: *‘Sometimes you might just ask the parent, how are you today? And that's it. It opens up a whole different thing that you didn't even know was even going on in their head’* **[N3]**.

Examples of support for parents came from a variety of sources including key nursing staff and allied health professionals such as speech and language therapists and occupational therapists, for example: *‘So we're very lucky actually, we have a lot of different things going on.….we have coffee afternoon, that's run by our occupational therapist and sometimes some of the nurses ….the parents can go and they have a cup of tea and a chat, essentially with other parents’* **[N3]**.

All participants raised the importance of early bonding experiences and skin‐to‐skin time but thought that these strategies were necessary so that parents could learn the tools to confidently develop other care skills. They were considered important primary activities that would give a baseline for introducing and developing other caring abilities, such as training parents *‘how to do even nasogastric tube feeding’* **[N5]** or developing competent skills with expressing milk, *‘one thing we can do is really have them to express the feeding….That's ……… how they feel connected to the baby’* **[N6]**. The initial nursing process involved observing care, prompting as well as allowing parents to verbalise their concerns about learning to care for their preterm infant so they could *‘look after their baby and get used to their baby in the situation that they're in’* **[N3]**. There was an acknowledgement that parents were dealing with their own stresses of everyday life whilst simultaneously learning to look after their infant. This complex dynamic could impact parent emotional states. All participants understood the multifaceted needs of families when preterm infants were first admitted realising that *‘parents will be in a shock state’* **[N6]**, not knowing what to expect generally or what the specific needs of an infant born preterm would be. Personal problems such as *‘housing issues’* **[N2]** could impact on a parent's ability to develop infant care skills. Other influences could be the unfamiliarity of the neonatal environment, including barriers perceived to prevent bonding such as the incubator which *‘looks very medicalised and scary’* **[N3]** alongside the lack of privacy *‘because obviously they're in a room of 6 or 7 other babies and there's 6 or 7 other parents and they maybe feel like people are listening or, I don't know, I'm sure it can be quite difficult’* **[N3]**. To add to these barriers, nurses reported that parents of infants in neonatal intensive care can find the environment highly stressful, with potential negative impacts on nurse–parent interaction:

*‘The communication, trying to get them to communicate is a lot, I feel is a lot more difficult compared to the other rooms. Just purely because of all the sort of emotions, and how busy we are, and you know, so baby is really, really, really unstable and really sick, we're not, you know, it's not the sort of best thing that we're thinking of’*. **[N4]**.

There was recognition that fathers could be vulnerable to exclusion from the opportunity to bond and develop an understanding of their infant, as they were likely to still be working and only able to come *‘late in the evening or maybe just on the weekend’* **[N5]**. It was felt important *‘to make sure the father feels relaxed as possible and make sure that they feel like everyone's in safe hands and that they can go to and from freely and give them information’* **[N3]**. One nurse reflected that for some families, spiritual needs could be important to encourage to develop confidence with caring for their infant and as a method of providing some emotional support:

*‘Why, don't you try to read them a little bit of a piece of the Koran, one of the pieces (sic) of the Bible, or whatever is important to you’* **[N2]**.

3. Encouraging communication strategies: ‘We've just got to reiterate to the parents that they do know that you're there and that they want to communicate back with you’ [**N3**]

‘Communication’ was interpreted as either meaning discussion between staff and parents to share information, or as developing a constructive interaction between infants and their parents. Developing a positive staff–parent dynamic was considered essential within the neonatal nursing role, in conjunction with allied healthcare professionals; for example, speech and language therapists, occupational therapists and psychologists: *‘We spend a lot of time initially when babies are first admitted……communicating to the parents sort of the information about what's happening and what they can do for their baby’* **[N3]**. There was an awareness of the stress experienced by families when first admitted to a neonatal unit, and that *‘*….*every parent is different’* **[N7]**, with some able to cope with information and others finding the experience *‘overwhelming’* **[N7]**:

*‘I don't know, when they see a premature baby they think, oh they can't hear me or they can't, do you know they can't feel that I'm there and they feel very like disconnected because they're plunged into this really foreign environment which wasn't what they were expecting’* **[N3]**.

Direct information sharing with parents, both face‐to‐face and via technology such as WhatsApp, from admission to the unit and post‐discharge, was seen as necessary:

*‘The parents need a clear picture of what's going on ‘cause they will be really confused………even if we explain in the beginning that's(sic)just going to happen. But they'd be asking repeating the same questions again and again, ‘cause they don't understand the situation’* **[N6]**.

In relation to communication with the infant, many nurses referred to infant behavioural characteristics and cues, rather than specific examples of functional strategies that provide a scaffold for the development of speech, language and communication. Most participants described communication in terms of breastfeeding, listening to parent and family voices, bonding and skin‐to‐skin time. One **[N9]** mentioned that for parents and infants to develop a mutually satisfactory communication and interaction dynamic, she *‘would recommend them to talk to the babies………to suggest talking when they touch them’* and that eye contact was necessary between the two who were interacting **[N9]**. Encouraging infants to begin *‘communicating with their parents from the get‐go’*, especially during feeding, was seen as another method of developing parent confidence **[N7]**.

Other approaches considered as communication included recommending that parents *‘take small, small simple baby books’* **[N9]** to share, or using *‘a soft voice’*, try to *‘dim the lights’* and singing **[N4]**. Others commented that they encouraged parents to talk to their infants about what they were doing during care **[N1]** or read or sing so that the parent's voice could be heard:

*‘But then in terms of communication we do try and like promote, well try and ask mum and dad to sort of sit with the baby and talk to the baby, have books as well which they… I think they've been removed actually, haven't they but we did have books because sometimes I feel like they feel a bit awkward in what to say, in the sense that everyone else is there …’* **[N3]**.

*‘We encourage mums to talk to their baby because a parent's voice is the best’* **[N5]**.

*‘And if mommy is happy talking to people and talking to their baby, they will be happy to listen to those voices’* **[N2]**.

Barriers that could delay the development of parent communication confidence could be due to a *‘language barrier’* **[N7]** and the fact that English was an additional language **[N1, N3],** impacting the ability to clearly share a rationale for developing early infant language interaction. Personality was mentioned as a factor that could slow down the development of infant communication **[N7]**, including being overwhelmed with the *‘busy‐ness of Room 1….because there's just so much going on’* **[N4]** and *‘*little privacy*’* **[N8]**. The need for an infant to be in an incubator and/or infant illness could prevent or limit early communication strategies and prohibit parental confidence–building skills **[N5; N8]**:

*‘What I've seen, for example, is when the baby is born premature some parents will be so scared not to get involved and they don't get involved and because the baby's so fragile they might think that the baby probably can't, it's? a difficult journey and they can't make it, so they….stay away because of the fear that they might lose the baby and actually bonding more will make it harder for them’*.**[N5]**

Parents were regarded as experts in talking directly to their infants with differences noted between infants visited regularly compared with those who had fewer family visits:

*‘I mean, early communication is vital because we see the difference from the baby that doesn't have the parents as much with a baby that has got mum there all the time’* **[N5]**.

Other benefits of encouraging communication with an infant included a description of nursing an infant with chronic lung disease who was experiencing oxygen desaturation, with the suggestion that the use of voice could help stabilise them. One nurse mentioned that the mother could use her voice to *‘make a story with the…… sibling….and actually, it did help’* **[N2]**. Sometimes, recognition was given to the fact that parents need *‘the opportunity to be able….to feel comfortable’* **[N7]** in developing a speaking and communication style with some parents modelling for others:

*‘They sing and they talk, and they do it straight away for other parents’* **[N7]**

4. The impact of limiting parental presence and wearing facemasks: ‘When they had to skin‐to‐skin, they were asked to wear the facemask, and they really couldn't see the baby’ [**N6**]

Discussion about communication prompted participants to reflect on the reduced opportunities for interaction during the COVID‐19 pandemic, which was a significant part of their narrative. From this, as well as the subsequent impact on daily exchanges with peers, parents, and infants, there was a strong sense that reduced parental access to neonatal units negatively influenced infant development, and specifically, without doubt, communication:

*‘Babies didn't progress as fast as they could have with the parents in the unit. Each parent on the unit, baby will do breastfeeding, mummy will understand the baby. Babies progress quicker when the mums are there’* **[N5]**.

*‘See how these babies that come through that? Are they going to grow up? What's their communication going to be like, because it's all masked…wonder how much that's gonna have impacted them as they grow up’* **[N8]**.

All participants shared examples highlighting the impact of limited parental access on parent confidence and well‐being. Time away from infants was seen as detrimental to development, and there were concerns about parents’ ability to learn and understand their babies:

*‘That child was just constantly crying…attention seeking for a month, because everybody else was a stranger…so it took a lot longer to get him to accept even his grandparents’* **[N8]**.

*‘When there was really restricted visiting…I think that really impacted on the babies. Some were COVID positive and there'd be 2 weeks when a parent couldn't come in, so we'd have to send videos, using vCreate’* **[N3]**.

While some support was offered via technology (like vCreate) to update parents on infant development, many noted the limitations of video‐based methods. These were seen as brief, lacking context and unable to replace in‐person involvement:

*‘We have vCreate…we record a quick snapshot of the baby and send it. COVID time was like we had the unit all only babies and no parents’* **[N5]**.

*‘[vCreate] we recorded kind of like 1 minute. It couldn't be as long as you like’* **[N7]**.

There was also shared empathy and sadness among staff as they recalled moments when parents were unable to be with their infants:

*‘We did a lot of bath times [using vCreate] or if in a side room, we'd show them around. Just so the parents knew where their babies were. But they couldn't grasp how big they were…we tried to put things next to them so they could gauge their size. It's a two‐way communication. It's not live, but they can send videos in too…we were all bawling our eyes out with this’* [**N7]**.

The re‐introduction of in‐person visiting was welcomed, especially the return of siblings and extended family, with nurses acknowledging the social and interactive benefits of wider familial involvement:

*‘We have just re‐started siblings visiting because we didn't allow them before’* **[N9]**.

*‘Siblings are now allowed back…they just chatter, chatter, chatter to the baby. It's so sweet…It's very important to get that first part of communication early’* **[N3]**.

Face masks were widely described as a barrier to communication, both among staff and between parents and infants. The inability to read facial expressions was a concern for both safe practice and bonding:

*‘It's impacted us speaking to one another…can't see facial expressions, can't see how someone's reacting to the conversation’* **[N1]**.

*‘We are more prone to make mistakes because we cannot see each other's faces. I'm a foreigner…how I express myself might be misinterpreted. Even if I mean well, the parent could interpret it differently’* **[N2]**.

Although necessary for safety, masks were described as detrimental to the experience for both parents and infants:

*‘It was not a very good experience with the parents’* **[N6]**.

*‘Parents said, “My baby doesn't smile at me”’* **[N5]**.

Nurses observed that infants showed unfamiliarity and surprise when finally seeing unmasked faces:

*‘Then you take your mask off and smile, and they just open their eyes so wide because they're not used to it…Then you move your hands and say hello, and they just don't know what to do. I think there's going to be a developmental impact’* **[N2]**.

*‘Facemasks are a big barrier. Facial expressions are a huge part of communication. I try to overcompensate, because I know half my face is covered’* **[N4]**.

Challenges in teaching parents about infant care and bonding were exacerbated by reduced in‐person time. Nurses expressed concern about limited opportunities to provide guidance, especially in preparation for discharge:

*‘There's not much [online] about the development of communication’* **[N8]**.

Face‐to‐face interaction was seen as vital for understanding both parent and infant needs. One nurse reflected on the importance of in‐person visits:

*‘I wasn't planning to see a parent because I thought the baby was well…after listening to mum, I thought she sounded too quiet…So I went down, just to get more of a feel for what was going on’* **[N8]**.

Finally, restrictions even extended to critical early care: *‘When I was in X during full COVID, we didn't even do breastfeeding. If you think about it, it's crazy’* **[N2]**.

## 5 Discussion

This narrative inquiry study examined neonatal nurses’ experience of working in neonatal care to gain their insight into the factors that can enhance or hinder early infant–parent communication within a neonatal unit, along with the part they play within this vital area.

Participants discussed the intense and necessary support that parents require when their infant is first receiving neonatal care; developing communication with an infant was harder if they displayed unstable health. The neonatal unit is known to be a new and challenging environment for families to adjust to whilst also trying to acquire new skills (such as the giving of daily care, pre‐feeding and feeding abilities, developing ways to bond and connect confidently with their new and vulnerable infant) and, at the same time, trying to understand their medical needs (Cardoso et al. 2015). Green et al. (2021) recognise that parents need time to cope with new experiences and to develop connections with their infants as they become familiar with the neonatal unit setting; these parents value specific guidance from nursing staff (Guillaume et al. 2013). Equipment, such as an incubator, can pose barriers to developing care skills early in an infant's life (Coppola and Cassibba 2010), but support from nursing staff can help parents to develop confidence (Muller‐Nix et al. 2004).

Unmanaged parent stress can impact all the essential early skills that enable parents to bond with and care for their child (Korja et al. 2008). Nurses recognise the substantive stress that parent's experience, in particular balancing home, work and neonatal unit commitments. A systematic review and meta‐analysis of strategies that neonatal nurses provide for families (Malaki et al. 2022) identified that emotional support, help with mother–infant attachment, maternal empowerment and practical modelling that promotes confident and direct infant care are essential; of the papers evaluated in the review, all intervention groups where nursing support was provided, reported significantly lower levels of stress in mothers (Malaki et al. 2022). Participants in our study used a diverse range of approaches to enable the introduction of a rich communication setting for infants. Neonatal nurses are in an ideal position to encourage parents to develop confident interactions with their infants as well as identify the relevant professionals who can provide the necessary resources to enhance experiences (Gibbs et al. 2021).

Our participants recognised the importance of supporting and helping parents to bond with their preterm infant, usually through close skin‐to‐skin time, or more specifically through daily care interventions such as naso‐gastric tube feeding, expressing of breast milk and direct feeding. These examples were frequently referred to as *‘communication’* but interestingly, none of the nurses discussed the benefits of extending these important skills to develop meaningful linguistic interactions. A wide range of communication strategies mentioned in the study included observing infant behaviours, allowing infants to hear family voices, touching and talking, singing and reading quietly to an infant. Saliba et al. (2018) highlight the importance of the parent voice in providing infant stability with measures such as heart rate, oxygen saturation, respiratory rate and behavioural responses being positively impacted; this reinforces the benefits for infant health in both the immediacy and longer term.

All participants recognised ‘*communication*’ within the neonatal unit as being vital, for both their direct work with infants and when supporting parents. The fact that infants born preterm are at significant risk of developing speech, language and communication problems means there is a need to encourage early language‐rich experiences to try to minimise poor language outcomes; however, this aspect was not mentioned by any of our participants. Only one nurse commented on an essential non‐verbal requirement for developing sustained interaction, that being eye contact. Knowledge of the importance of using specific and repeated vocabulary to help structure early language learning is essential; Caskey et al. (2014) demonstrated that infants receiving adult word exposure at 32 and 36 weeks in a neonatal unit had better language outcomes on discharge and when re‐assessed at 7‐ and 18‐month corrected age. Education about how to use specific vocabulary when establishing a positive communication dynamic is important for all neonatal staff, particularly nurses, as they tend to use a low count vocabulary when interacting with infants (for example, talking less when a painful procedure is being undertaken) (Zwimpfer et al. 2021). English is an additional language for some parents, so there is a need to provide nurses with training about how to provide inclusive, positive and enriched early bilingual communication environments.

All participants referred to the impact of COVID‐19 and the influence on both parent stress and infant development. The intense use of facemasks drew attention to the importance of facial expression during the development of interactions. Facemasks were also associated with less vocalisation by adults. Nurses felt that the reduced neonatal unit access that parents had during the pandemic, along with limited infant views of people's faces, delayed infant development. Language‐rich environments are known to have benefits for children as they develop, including those born preterm (Rabie et al. 2015; Sanchez et al. 2019). Specifically, delays were experienced when responding to infant emotions as parents were not present; use of vCreate captured infant progress but was not felt to be the same as parents being present. Harding et al. (2022) identified the core skills required for communication, which included eye contact, initiating and responding to vocalisations, use of natural gestures, vocal intonation and intensity and facial expression; related and supportive adjunct communication skills included singing and reading. At present, it is unclear if reduced neonatal access and use of facemasks has had a negative influence on the language development of the COVID‐19 cohort of neonatal unit infants; this group was already prone to delayed language and cognitive skills that may impact learning and social development at school (Imgrund et al. 2022) so time will reveal any further effect on their health and well‐being. However, it seems likely that the use of PPE will have inhibited natural and spontaneous communication such as natural gestures, facial expression and the amount of language that might have otherwise been spontaneously initiated by nurses (Iverson and Goldin‐Meadow 2005).

### 5.1 Strengths and Limitations

A key strength of this study is that a unique insight into the experiences of neonatal nurses was provided. The same member of the research team conducted all the interviews and was not known to the neonatal units where the investigation was undertaken. All three researchers were experienced in qualitative research methods and familiar with the analysis of narrative inquiry–generated data; this enhanced the overall rigour and trustworthiness of the study. Interpretation of data and identification of themes were discussed and agreed. All three researchers have had experience of caring for neonates within an intensive care environment; this facilitated understanding of the nurses’ narratives and their perspectives. As seen in Table 1, we were able to recruit a diverse sample from five different ethnicities with nurses having a variety of roles and lengths of service.

Regarding the study limitations, the neonatal units were within the same NHS Trust, so this may have meant that participants vocalised similar opinions. The two units were dependency Levels 1 and 2; therefore, it was not possible to explore experiences in a Level 3 area (higher dependency level). In addition, although several nurses had previously worked in a range of other neonatal units, with previous experiences adding to the richness of the data, it was not evident that they had worked in Level 3 neonatal units; this perspective would be useful to add to the overall picture. Our sample size was small with all participants identifying as female—however, this is reflective of the predominately female neonatal nursing population within the United Kingdom and is in line with other narrative inquiry work that seeks to explore neonatal nursing perspectives (Hall et al. 2010; Gibbs et al. 2021; McKenzie et al. 2021). Moreover, our clear audit trail will facilitate transferability.

### 5.2 Recommendations for Future Research

Gauging the views of a larger group of nurses from an increased range of neonatal units, where there may be different allied health professional support, would yield useful comparisons with our narrative inquiry study. We did not interview any neonatal nurses from a Level 3 neonatal intensive care unit, and we would recommend that this is included in future work.

As well as the staff perspective, parents’ views are also essential in understanding infant communication development. Whilst some research has been undertaken which highlights both the barriers and positive experiences of supporting parents to develop early communication strategies within a neonatal setting (Petty et al. 2024), research that includes a paternal perspective (such as Romeo et al. 2023) does not have a UK context—we suggest that this warrants further investigation.

The linguistic analysis of interactions between infants and parents during different daily care activities, as well as at varying stages of an infant's development, would be useful to gain a richer understanding of the range of language and communication styles used. This data would enable additional resources and strategies to be more carefully tailored to meet the needs of preterm infants and their families to enable quality interaction to be integrated into neonatal care and their usefulness to be evaluated.

### 5.3 Implications for Policy and Practice

This study has highlighted that there is a need to improve nursing knowledge of early infant–parent communication development and interaction, including an understanding of communication facilitation when English is not the first language used by a family. Beyond skin‐to‐skin care and interpretation of infant behaviours, early communication and interaction needs to include the essential components of gaze, sustained eye contact, gesture, vocalisation, use of gesture to support simplified language and responsiveness as well as reciprocity within a linguistic context (Harding et al. 2022). A systematic review of direct early infant communication within a neonatal environment identified only five papers; in contrast, there is a wealth of literature that investigates early infant–parent bonding techniques that often describe their methods as promoting ‘communication’ (Harding et al. 2019).

Preterm birth is a risk factor for a range of longer‐term problems, including language development, which can have an impact on educational achievements and the development of friendships in school (van Noort‐van der Spek et al. 2012). Therefore, it is essential that clearer and more easily available resources, which highlight the associated speech, language and communication risks, are available to neonatal staff so that they can support parents appropriately. These need to be distributed more directly by key professionals, such as speech and language therapists who regularly collaborate with neonatal nursing staff.

The educational focus for nurses is vital; there are significant increases in the risk of mild or moderate language impairment in early preterm children (<27 weeks) in comparison to term infants at 2.5 years of age (Serenius et al. 2013). Very preterm infants (≤25 weeks) have a high risk of developing pervasive and persistent speech, language and communication problems (Wolke et al. 2008) with additional risks that inhibit effective language acquisition such as less use of gesture (Sansavini et al. 2011), difficulties developing the ability to link words effectively (Iverson and Goldin‐Meadow 2005), delayed acquisition of first words, difficulties with grammatical rules, shorter sentences, as well as fewer nouns and verbs within expressive language repertoires (Sanchez et al. 2019). Aside from the expressive language deficits, children born preterm are also at risk of receptive language (understanding of language) problems (van Noort‐van der Spek et al. 2012).

Encouraging early interaction that integrates effective and supportive strategies during infant care activities will not prevent speech, language and communication difficulties from emerging, but can maximize an infant's language learning opportunities and empower parents to establish quality inclusive interactions that can be extended post‐discharge. We advocate the need for nurses to understand the development of language in a multi‐lingual household and how to confidently support parents to interact with their infant when English is an additional language.

Future research is needed to inform the education of neonatal practitioners in relation to relevant communication strategies that can enrich early dyadic synchrony during the interpretation of infant states and when observing and responding to homeostatic regulation. Speech and language therapists already work in neonatal units supporting both infant feeding and early communication whilst working closely with neonatal nurses, multi‐disciplinary team members and parents; they are ideally placed to work with other neonatal professionals to promote strategies that can provide an enriched communication setting (Harding et al. 2022). Due to the substantive risks that may impact preterm infant development, it is imperative that any education and future research focuses on developing and promoting strategies to support early speech, language and communication strategies for both nursing staff and families within neonatal units.

## 6 Conclusion

Neonatal nurses have complex and challenging duties to undertake when working with preterm infants and their immediate families; they recognise that encouraging parents to bond, touch, hold and interact with their infant is important, but their knowledge related to specific approaches in relation to developing an early linguistic framework is limited. It is suggested that further work, developed by speech and language therapists collaborating with neonatal nurses, could promote early communication strategies within neonatal care. This recommendation is relatively low cost, but when used with appropriate, sensitive and regular modelling and education, it can be hugely beneficial to both parents and infants.

## Consent

This study did not require patient consent. Nurses were recruited to take part in this study. Our study adhered to strict research protocols in which information about the study was given, opportunity for questions pre‐participation was encouraged and signed consent was gained. The study was approved by both the first author's University and from the Research Committee of the NHS Trust (IRAS ID: 309641) where the research took place.

## Conflicts of Interest

The authors declare no conflicts of interest.

## Permission to Reproduce Material From Other Sources

This study did not reproduce material from other sources.

## Supporting information


**Supporting File**: jlcd70093‐sup‐0001‐SuppMat.docx

## Data Availability

The data associated with this study are not publicly available. However, data access can be requested from the corresponding author on reasonable request.

## Topic Guide

Exploring neonatal nurses’ and parents’ understanding of the factors that enhance and hinder communication and early interaction between preterm infants and their parents

The broad aim of this interview is:

To gain insight into your knowledge and experiences relating to early infant communication in the neonatal unit during your time working in this area.

Key (SQIN*) Question:

*Thinking about your time working on the neonatal unit, could you tell me how you have supported parents to communicate and interact with their preterm babies? You can draw on examples as you talk this through*….(*SQIN‐ Single question to induce narrative)

These additional questions/points of interest may be prompted through the interview depending on what the participant says for the first opening question……

• *To give us some background, could you tell me about your neonatal care background, how long you've worked here etc…? / (ie. Talk me through from the start to the current day]*
*• Can you tell me (a bit more) about the types of babies you commonly care for, along with their parents. and the type of support these families need?*
*• Do you think early communication & interaction between parents and their preterm babies is important and if so, why?*
*• What do you see as your role in relation to parent‐infant communication, specially relating to prematurity?*
*• Was / Is there anything in particular that helps parents communicate with their preterm baby? (give examples)*
*• Were / Are there any particular challenges for parents that you have observed in relation to communication—for example, any barriers, times when communication is difficult, other factors that hinder communication?*
*• Can you explain how you would know if it is the right time for parents to communicate with their preterm baby?*
*• Have any previous experiences either helped or hindered how effectively you support parents to communicate with their preterm baby?*
*• What support services are offered relating to parental support with communication?*
*• What resources would be useful to assist you in supporting parents to communicate / interact with their preterm baby?*
*• Is there anything in particular that you think a health professional could/should offer in order to support parents better to communicate / interact with their preterm baby?*
*• Is there anything else you would like to add in relation to communication on the neonatal unit?*
