# Supplementary material for: Neonatal Nurses’ Understanding of the Factors That Enhance and Hinder Early Communication Between Preterm Infants and Their Parents: A Narrative Inquiry Study
Source: Int J Lang Commun Disord. 2025 Jul 14;60(4):e70093. doi: 10.1111/1460-6984.70093 (PMC12257063; doi:10.1111/1460-6984.70093)
Supplement: Supplementary file 1 — Supporting File: jlcd70093‐sup‐0001‐SuppMat.docx [file JLCD-60-0-s001.docx]

Consolidated criteria for reporting qualitative studies (COREQ): 32-item checklist

**Neonatal nurses’ understanding of the factors that enhance and hinder early communication between preterm infants and their parents: A narrative inquiry study**

| **No** | **Item** | **Guide questions/Description** |
| --- | --- | --- |
| Domain 1: Research team and reflexivity |  |  |
| Personal characteristics |  |  |
| 1. | Interviewer/Facilitator | Which author/s conducted the interview or focus group?  *1st author––Dr Julia Petty* |
| 2. | Credentials | What were the researcher's credentials? *For example, PhD, MD*  *All authors have PhDs and are at professor/associate professor grades.* |
| 3. | Occupation | What was their occupation at the time of the study?  *Dr Julia Petty––Associate professor/Dr Lisa Whiting––Associate professor/Dr Celia Harding––Honorary professor* |
| 4. | Gender | Was the researcher male or female?  *All female* |
| 5. | Experience and training | What experience or training did the researcher have?  *We all have extensive qualitative research experience having led and been involved in previous research studies over the past 10 years at least including doctorate studies and supervision of doctoral students ourselves. This has involved training in research methods, ethics and reflexive approaches.* |
| Relationship with participants |  |  |
| 6. | Relationship established | Was a relationship established prior to study commencement?  *The participants were not known to the researchers.* |
| 7. | Participant knowledge of the interviewer | What did the participants know about the researcher? For example, *personal goals, reasons for doing the research*  *They knew about the aim and purposes of our study and our background, place of work as researchers.* |
| 8. | Interviewer characteristics | What characteristics were reported about the interviewer/facilitator? For example, *bias, assumptions, reasons and interests in the research topic*  *Transparency was demonstrated in the interview initial brief and prior to the interview––The researchers were open about the aims of the research, and this was all stated in our detailed participant information sheet that was disseminated to the participant at the point of recruitment.* |
| Domain 2: Study design |  |  |
| Theoretical framework |  |  |
| 9. | Methodological orientation and Theory | What methodological orientation was stated to underpin the study? *For example, grounded theory, discourse analysis, ethnography, phenomenology, content analysis*  *A narrative inquiry approach was used for the study.* |
| Participant selection |  |  |
| 10. | Sampling | How were participants selected? *For example, purposive, convenience, consecutive, snowball*  *Purposive sampling* |
|  |  |  |
| 11. | Method of approach | How were participants approached? *For example, face-to-face, telephone, mail, email*  *A recruitment poster was disseminated to the relevant trusts/neonatal units and email correspondence via specific gatekeepers (consultants and managers of the neonatal units within two NHS trusts).* |
| 12. | Sample size | How many participants were in the study?  *Nine neonatal nurses* |
| 13. | Non-participation | How many people refused to participate or drop out? Reasons?  *No participant dropped out. This was on a volunteer basis, so once the participants came forward, they continued with the interview.* |
| Setting |  |  |
| 14. | Setting of data collection | Where was the data collected? For example, *home, clinic, workplace*  *Remotely via Zoom teleconferencing* |
| 15. | Presence of non-participants | Was anyone else present besides the participants and researchers?  *No* |
| 16. | Description of sample | What are the important characteristics of the sample? *For example, demographic data, date*  *Inclusion criteria were applied––Neonatal nurses currently working within a neonatal unit within one of the two trusts who took part in the study. Exposure to direct contact with parents of preterm infants in their workplace was required.* |
| Data collection |  |  |
| 17. | Interview guide | Were questions, prompts, or guides provided by the authors? Was it pilot-tested?  *An interview guide was produced (using a narrative open question and then prompts), which went through full ethical approval and a parent advisory group was involved in the question development.* |
| 18. | Repeat interviews | Were repeat interviews carried out? If yes, how many? *No* |
| 19. | Audio/Visual recording | Did the research use audio or visual recording to collect the data? *Yes, audio recording was used with consent.* |
| 20. | Field notes | Were field notes made during and/or after the interview or focus group?  *No, this was not deemed necessary and would have detracted from listening and giving the participant due attention.* |
| 21. | Duration | What was the duration of the interviews or focus group?  *Between 40 and 55 min each* |
| 22. | Data saturation | Was data saturation discussed?  *Yes, we called this ‘information power’––Nine nurses were deemed sufficient on discussion to yield rich data.* |
| 23. | Transcripts returned | Were transcripts returned to participants for comment and/or correction?  *This was offered to the participants but none expressed a wish to have the transcripts returned to them. We left this to choice.* |
| Domain 3: Analysis and findings |  |  |
| Data analysis |  |  |
| 24. | Number of data coders | How many data coders coded the data?  *Two. Dr Lisa Whiting and Dr Celia Harding. Independent coding was done, followed by discussion and agreement.* |
| 25. | Description of the coding tree | Did authors provide a description of the coding tree?  *No, but we did not feel this was needed and would not add anything to the study write-up. We also used a narrative design, which does not directly employ strict coding per se––It is a more fluid and open approach to analysis using a reflexive approach.* |
| 26. | Derivation of themes | Were themes identified in advance or derived from the data?  *Derived from the data* |
| 27. | Software | What software, if applicable, was used to manage the data?  *No specific software was utilised.* |
| 28. | Participant checking | Did participants provide feedback on the findings?  *Not at this stage, although they have access to the final report and will be sent the final paper.* |
| Reporting |  |  |
| 29. | Quotations presented | Were participant quotations presented to illustrate the themes/findings? Was each quotation identified? For example, *participant number*  *Yes, quotations were used with anonymous identification (by a number)* |
| 30. | Data and findings consistent | Was there consistency between the data presented and the findings?  *Yes* |
| 31. | Clarity of major themes | Were major themes clearly presented in the findings?  *Yes* |
| 32. | Clarity of minor themes | Is there a description of diverse cases or a discussion of minor themes?  *Sub-themes were discussed under each theme heading. No diverse cases were identified. Limitations in relation to the diversity of the sample were acknowledged in the Discussion.* |
